# Disparities in treatment burden in Peru among people with multiple long-term conditions by geography and healthcare facility: A cross-sectional study

**DOI:** 10.1371/journal.pgph.0007355

**Published:** 2026-09-29

**Authors:** Silvana Perez-Leon, Daniel Mendoza-Quispe, Maria Sofia Cuba-Fuentes, Juan Carlos Bazo-Alvarez, M. Amalia Pesantes, Andrea Gaspar, Joseph R. Zunt, Victor M. Montori, Jose Armando Castillo, Eduardo Carcausto, Nelson Diaz, Keveen Salirrosas, Pilar Cornejo, J. Jaime Miranda

**Affiliations:** 1 CRONICAS Center of Excellence in Chronic Diseases, Universidad Peruana Cayetano Heredia, Lima, Peru; 2 Department of Medicine, Faculty of Medicine, Universidad Peruana Cayetano Heredia, Lima, Peru; 3 Center for research in Primary Health Care, Universidad Peruana Cayetano Heredia, Lima, Peru; 4 Research Department of Primary Care and Population Health, University College London, London, United Kingdom; 5 Department of General Medicine, University of Pittsburgh Medical Center, Pittsburgh, Pennsylvania, United States of America; 6 Departments of Neurology, Global Health, Medicine (Infectious Diseases), and Epidemiology, University of Washington, Seattle, Washington, United States of America; 7 Knowledge and Evaluation Research Unit, Mayo Clinic, Rochester, Minnesota, United States of America; 8 Faculty of Medicine, Universidad de San Martín de Porres, Lima, Peru; 9 Department of Internal Medicina, Clínica Internacional, Lima, Peru; 10 School of Medicine, Universidad Peruana Union, Lima, Peru; 11 Department of Medicine, Clínica Good Hope, Lima, Peru; 12 Corporate Medical Directorate, Clínica Médica Cayetano Heredia, Lima, Perú; 13 Department of Postgraduate Studies, School of Medicine, Universidad Peruana Cayetano Heredia, Lima, Peru; 14 Department of Endocrinology, Hospital Arzobispo Loayza, Lima, Perú; 15 Sydney School of Public Health, Faculty of Medicine and Health, The University of Sydney, Sydney, New South Wales, Australia; Sun Yat-Sen University, CHINA

## Abstract

People with multiple long-term conditions (MLTC) in low-resource settings face high treatment burdens, influenced by healthcare access and quality disparities. The study aimed to compare the treatment burden among patients with MLTC across geographical locations and healthcare providers in Peru. We conducted a cross-sectional study conducted between June–October 2023 among outpatients aged ≥40 years with type 2 diabetes and ≥1 additional chronic condition, recruited from public and private healthcare facilities in urban Lima and semi-urban Tumbes, Peru. Treatment burden was assessed using the Multimorbidity Treatment Burden Questionnaire (MTBQ). Differences in median MTBQ scores by setting and healthcare provider were analyzed using regression models adjusting for capacity- and workload-related variables. Among 555 participants (55% female, mean age 62.4 ± 10.5 years), median MTBQ score was 27.1 (IQR 14.6-41.7). After adjustment, patients in semi-urban Tumbes had higher treatment burden than those in urban Lima (adjusted median difference 6.1 points; 95% CI 2.9 to 9.3), while patients receiving care in private facilities had lower treatment burden than those in public settings (−6.6 points; 95% CI −9.3 to −3.9). These findings highlight substantial inequalities in treatment burden by place and provider type, with implications for efforts to improve care delivery and reduce disparities for people living with MLTC.

## Background

Multimorbidity, also referred to as multiple long-term conditions (MLTC) [1], is typically defined as the presence of two or more chronic conditions in an individual [1,2]. This growing phenomenon is of particular concern in low- and middle-income countries (LMICs) [3], where health systems face significant challenges in managing the increasing burden [4]. This differs conceptually from comorbidity, which refers to additional conditions relative to a defined index disease [5,6], whereas MLTC does not require a designated index condition. For individuals with MLTC, managing day-to-day life can become highly complex [7,8]. They must balance the demands of daily life with their available capacities [9], while also contending with the burden of illness associated with multiple conditions [10]. Additionally, this is compounded by the need to undertake a range of activities required to adhere to treatment regimens. These activities demand both workload and the capacity to carry them out by the patient but also within their social networks, a challenge which has an impact on their quality of life and is commonly referred to as treatment burden [7–9,11,12].

In LMICs, where social inequalities are more pronounced, healthcare systems are fragmented, and resources are limited, treatment burden is likely to be amplified. While the negative consequences of MLTC are well-documented, there is limited research examining treatment burden from MLTC in LMIC and specially in South America region. Public healthcare delivery systems in low-resource settings, often overburdened and under-resourced, likely place greater treatment demands on individuals, while rural settings present additional challenges due to limited healthcare access and resources [13].

Geographical location and healthcare provider type influence treatment burden in LMICs like Peru, as treatment burden is not experienced equally by all patients. Structural factors such as healthcare access, the quality of care provided, and geographic location are critical in shaping treatment burden [14,15].

We hypothesized that these factors are key drivers of treatment burden, with individuals in public and rural healthcare systems experiencing higher treatment burden compared to those in private and urban settings. This study aimed to explore these differences, focusing on assessing the association between geographic and healthcare provider factors with the treatment burden experienced by patients with MLTC in Peru.

## Methods

### Study design

Cross-sectional study (June 16th –28 November 2023).

### Study setting

#### Urban and semi-urban geographical areas.

This study recruited adults aged 40 or older receiving outpatient care from both private and public medical centers across two geographically distinct regions in Peru: Lima and Tumbes as shown in Fig 1.

**Fig 1:**
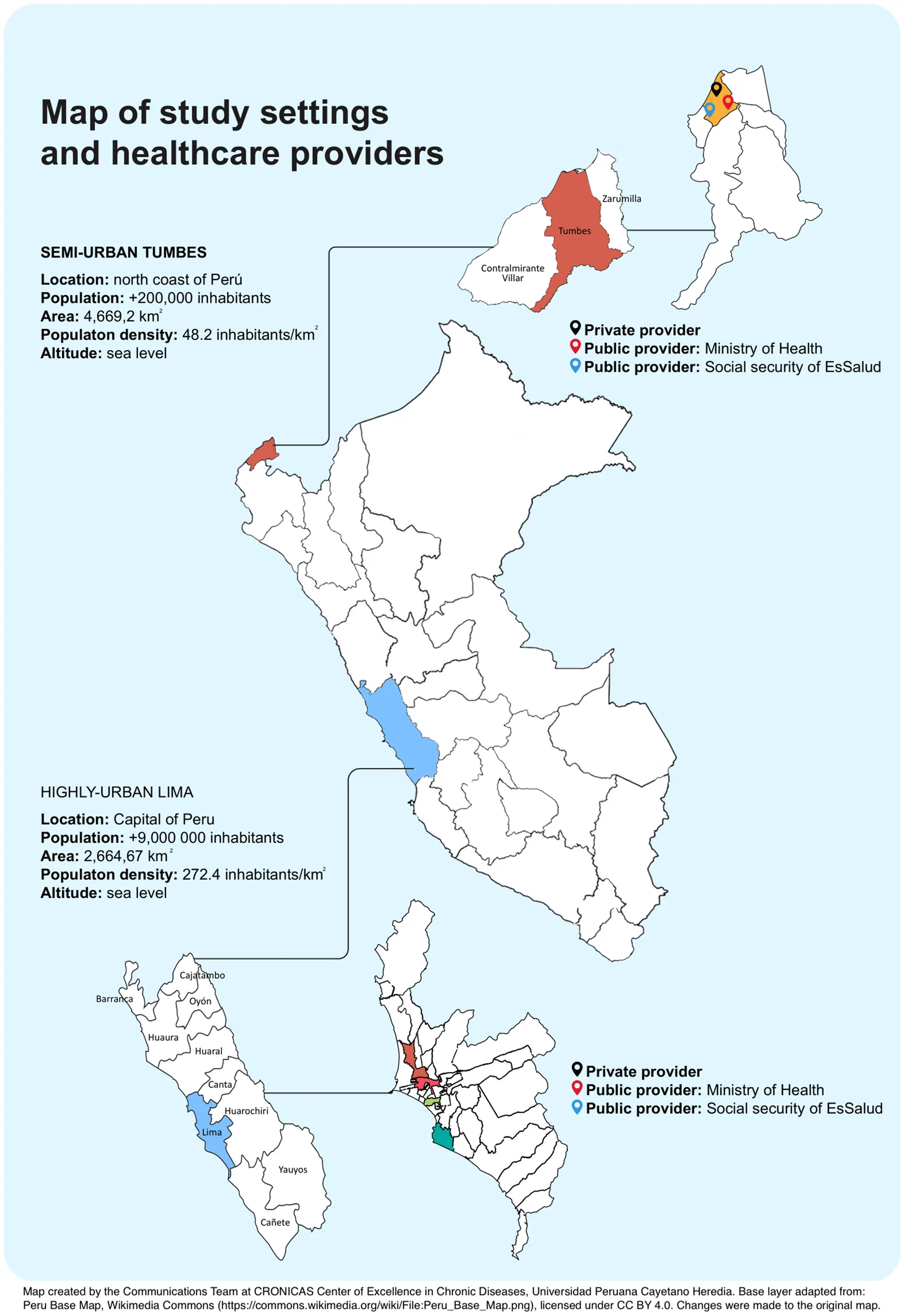
Map of study settings and healthcare providers. Map created by the Communications Team at CRONICAS Center of Excellence in Chronic Diseases, Universidad Peruana Cayetano Heredia. Base layer adapted from Peru Base Map, Wikimedia Commons (https://commons.wikimedia.org/wiki/File:Peru_Base_Map.png), licensed under CC BY 4.0. Changes were made to the original map.

Lima, Peru’s capital, is a highly urbanized city with over 10 million residents and a 2023 poverty rate of 29% [16]. It has a relatively well-developed healthcare infrastructure, with 440 inhabitants per doctor and 9,450 healthcare facilities [17,18].

Tumbes, a semi-urban northern region with over 200,000 residents and a similar poverty rate, has a less dense healthcare system, with 723 inhabitants per doctor and 112 facilities [17,18]. Selected for contrast, Tumbes borders rural areas, offering a contrast to the urban healthcare environment in Lima.

#### Public and private healthcare systems.

Peru’s healthcare system is supported by four main financing mechanisms [19,20]. The Ministry of Health (MoH) manages the tax-funded Seguro Integral de Salud (SIS) for low-income populations, while EsSalud, overseen by the Ministry of Labour (MoL), covers formally employed individuals. The military and police have separate insurance under the Ministry of Defense, and private insurance serves those opting for private care. Though each system has its own facilities, some overlap exists. This study recruited comparable numbers of patients from public and private healthcare centers in Lima and Tumbes.

Recruitment in Lima was conducted across four healthcare facilities, including two public institutions—one affiliated with the MoH and another with EsSalud—and two private clinics. In Tumbes, participants were enrolled through a combination of public healthcare facilities, representing both the MoH and EsSalud, and a network of private medical offices. Further description of these medical centers is provided in Table 1.

**Table 1:** Description of health providers.

	Complexity of care	Services offered	Number of inpatient beds
**Lima,** **highly urban**			
Public, Site #1, MoH, Hospital	Tertiary care (III-1)	Outpatient and inpatient	721
Public, Site #2, ESSALUD, Polyclinic	Primary care (I-3)	Outpatient	Not applicable
Private, Site #1	Secondary care (II-2)	Outpatient and inpatient	119
Private, Site #2	Secondary care (II-2)	Outpatient and inpatient	134
**Tumbes** **, semi-urban**			
Public, Site #1, MoH, Hospital	Secondary care (II-2)	Outpatient and inpatient	132
Public, Site #2, ESSALUD, Hospital	Secondary care (II-1)	Outpatient and inpatient	31
Private, Site #1, Clinic	Secondary care (II-E)	Outpatient and inpatient	5
Private, Site #2, Hospital	Primary care (I-3)	Outpatient	Not applicable

### Sample recruitment

Given the absence of comprehensive patient registries at the study sites, participants were recruited using a consecutive sampling strategy. Participants were eligible for this study if they had MLTC, defined as having type 2 diabetes and at least one additional chronic condition from a predefined list, which included hypertension, cardiovascular disease, cerebrovascular disease, tuberculosis, asthma, chronic bronchitis, chronic obstructive pulmonary disease, ischemic heart disease, depression, cancer, or other long-term conditions. Type 2 diabetes was selected as a tracer condition due to its high prevalence in LMICs, the significant treatment workload it entails, and its utility in benchmarking of health systems internationally [21].

Participants met the following criteria: (a)received care at the healthcare facility and lived in the geographical area for at least six months, (b)self-reported a physician diagnosis of type 2 diabetes with at least one additional chronic condition or related medication use for six months, and (c)provided informed consent. Participants were excluded if physical or cognitive limitations prevented them from participating in the survey or providing informed consent. Additionally, those with incomplete questionnaires (n = 6) were excluded from the analysis.

### Study procedures

Fieldworkers approached potential participants at outpatient consultations, informed them about the study, and confirmed eligibility before obtaining informed consent. Demographic and healthcare-related data were collected using password-protected electronic tablets. The MTBQ was self-reported or administered by fieldworkers for those with visual impairments or illiteracy (Text A and Text B in S1 Appendix). Participants received a type 2 diabetes self-care guide upon survey completion. Each site had four fieldworkers and a supervisor. Fieldworkers completed ethics training and 30 hours of in-person instruction before enrollment. For details in the study flowchart see Fig 2.

**Fig 2:**
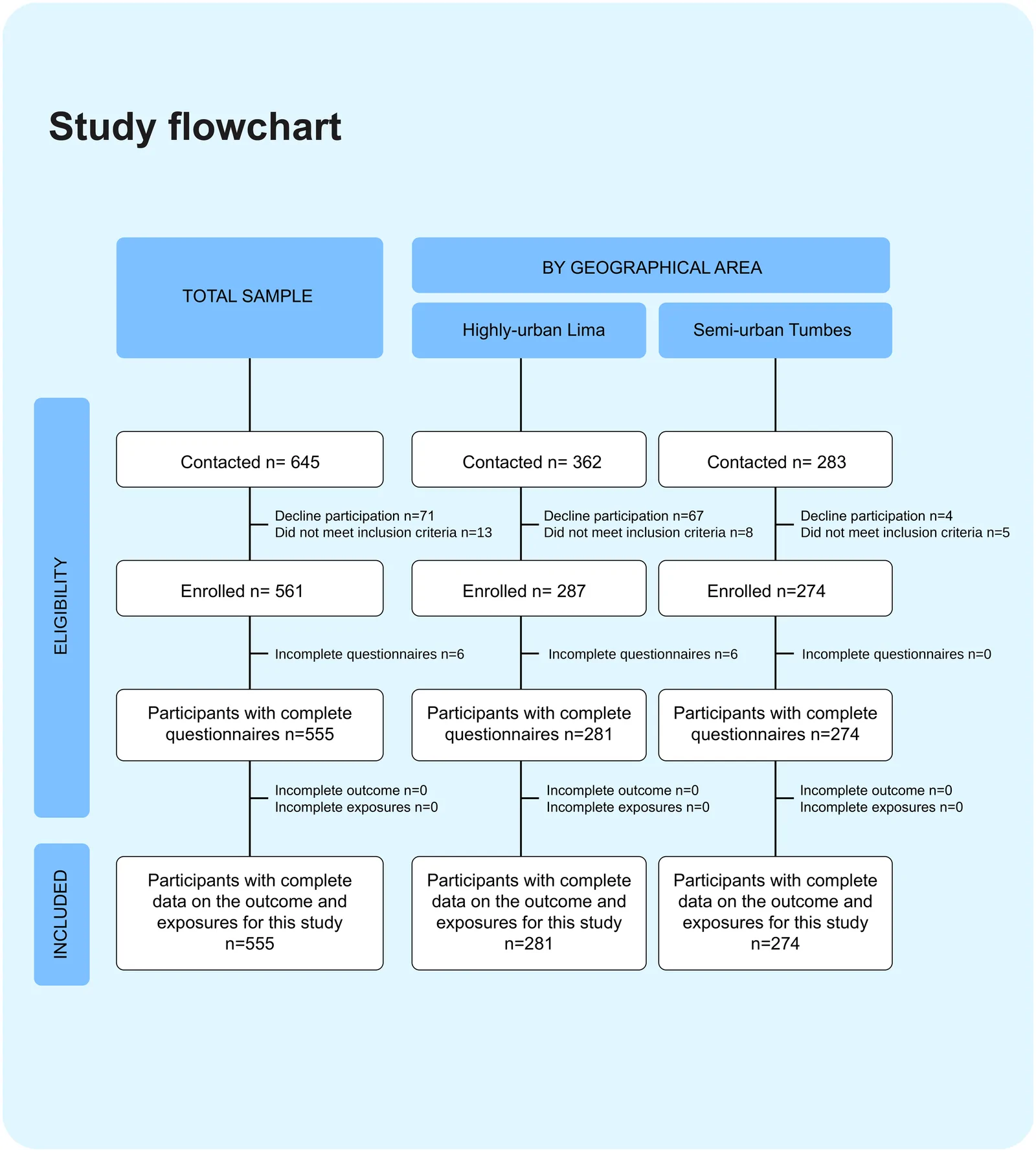
Study flowchart.

### Study variables

#### Outcome.

The primary outcome was treatment burden, measured using the MTBQ, a tool developed in the UK in 2018 for MLTC patients [22]. A Spanish version, S-MTBQ, underwent a comprehensive cross-cultural adaptation process prior to data collection, including forward and back-translation, focus groups for cultural validation, and a cognitive pilot test with 10 patients in Peru. Demonstrating good reliability (ω = 0.89 [95% CI 0.88–0.90]) and measurement invariance across geography (urban/semi-urban) and healthcare service (private/public); and convergence validity against measurements of depressive symptoms and adherence to treatment. Psychometric properties reported elsewhere [23]. The S-MTBQ consisted of 12 items, each scored from 0 to 4 points. A total treatment burden score was calculated by multiplying the mean score of all items by 25, resulting in a range of 0–100 points [22]. Further validation evidence of the MTBQ is summarized in Table A in S1 Appendix.

#### Exposures.

The primary exposures were geographical area and healthcare provider. Participants were classified as residing in a “highly-urban” area (Lima) or “semi-urban” area (Tumbes). Healthcare providers were categorized as public (government-funded) or private (independently operated).

#### Covariates.

We selected an initial set of sociodemographic, clinical, and healthcare-related variables based on a literature review of previous studies utilizing treatment burden measures. Sociodemographic variables comprised sex (female, male), age group (40–49, 50–59, 60–69, 70+ years), marital status (single [single, separated, divorced, or widowed] or partnered [married/common-law]), education level (primary or less, secondary, higher education), socioeconomic status (quintiles of a wealth index: lower, lower-middle, middle, upper-middle, upper) [24,25], self-identified ethnicity (mixed, quechua, white, other), and presence of a caregiver [26]. Clinical covariates included the number of self-reported chronic conditions [22,27,28], number of daily medications, insulin use, depressive symptoms (Patient Health Questionnaire-8, PHQ-8) [28–30], wheelchair use, outpatient consultations in the last year [24,25,31], and hospitalizations in the last year. Social, health and healthcare-related measures included family and social support as a proxy for social risk (Gijon’ Social-Familial Evaluation Scale) [32,33], quality of life (EQ-5D-3L [no problems, some problems, extreme problems]) [22,34–37], medication adherence (Adherence to Refill and Medication Scale, ARMS) [24,25,28,34,36,38], and patient’s report of receiving care that is congruent with the chronic care model (Patient Assessment of Chronic Illness Care [PACIC]) [24,39]. Definitions and further details on the interpretation of these measures are presented in Table B in S1 Appendix.

### Sample size

Previous studies reported mean MTBQ scores (±SD) of 13.5 (±16.1) [40], 14.5 (±15.3) [41], and 20.8 among patients with ≥2 or ≥3 chronic conditions [42]. For our study, we calculated the sample size needed to detect an average difference of at least 5 points in MTBQ scores between exposure subgroups. We assumed a type I error rate of 0.05, a power of 80%, and a standard deviation of 15.0. The required sample size was 426 participants (142 per subgroup in the worst-case scenario). To account for missing data, we increased the sample size by 15% to 489. Post-hoc sample size calculations by geographical area and healthcare provider are detailed in Table C in S1 Appendix, showing that the study is powered to detect ≥5 units of difference in the outcome.

### Statistical analysis

Data analysis was conducted using Stata V.16.0 (Stata Corp, College Station, Texas, USA). Sample characteristics were summarized overall and by geographical area and healthcare provider. Categorical variables were presented as absolute and relative frequencies, while numerical variables were reported as means with standard deviations (SD) or medians with interquartile ranges (IQR), depending on distribution. Characteristics of excluded versus included participants were compared to evaluate the impact of missing data.

We performed median regression (least absolute-value regression) with robust standard errors [43] to assess the association between exposure variables (geographical area and healthcare provider) and MTBQ scores. An incremental modeling approach was used: (i) Unadjusted model, to examine unadjusted associations between exposures and MTBQ scores, (ii) Capacity-adjusted model, adjusting for sociodemographic factors (sex, age, marital status, education, and socioeconomic status), which represent factors that have an impact in the capacity patients have to manage their treatment, and (iii) Workload-adjusted model, with further adjustment for clinical factors (caregiver presence, insulin and wheelchair use, outpatient consultations, hospitalizations, daily medications, and depressive symptoms) to account for treatment-related demands. Additional health and healthcare-related measures were not included in the adjustment as they were considered to lie on the causal pathway in the association of interest. Results were reported as beta coefficients with 95% confidence intervals (CI). We conducted additional post-hoc analyses by sequentially introducing variables from the workload-adjusted model, to identify the factor responsible for the difference between models. Essential analyses scripts are shared to facilitate analysis reproducibility (Text C in S1 Appendix).

### Ethics approval

All participants gave written informed consent, and data were de-identified for confidentiality. A master protocol was reviewed and approved by the Institutional Review Board of Universidad Peruana Cayetano Heredia as the main coordinating site, with additional approvals obtained at participating sites where ethics committees were available.

## Results

### Sociodemographic characteristics of the study sample

The analysis included 555 MLTC patients (55% female, mean age 62.4 ± 10.5 years), with 60% aged 60 or older. Six participants (1%) with incomplete questionnaires were excluded (Fig 2, Table D in S1 Appendix). Most participants (69%) reported both diabetes and hypertension among their chronic conditions, and an additional 54% were classified under “Other diseases” due to varied additional chronic conditions (Table E in S1 Appendix).

Table 2 presents detailed sample characteristics. Most participants were partnered (71%), while 14% reported having a caregiver. Only a third of the sample had completed education beyond high school. Socioeconomic disparities were more pronounced in public services and semi-urban Tumbes than in urban Lima.

**Table 2:** Study sample by geographical area and healthcare provider*.

	MTBQ score		Geographical area		Healthcare provider	
	Median (IQR)	Total N = 555	Lima N = 281	Tumbes N = 274	Public N = 365	Private N = 190
**MTBQ** **scores (tertiles**)						
Lower tertile (low scores)	11.5 (6.3-16.7)	200 (36.0%)	117 (41.6%)	83 (30.3%)	124 (34.0%)	76 (40.0%)
Mid tertile	27.1 (25-31.2)	172 (30.1%)	74 (26.3%)	98 (35.8%)	104 (28.5%)	68 (35.8%)
High tertile (high scores)	47.9 (41.7-56.2)	183 (33.0%)	90 (32.0%)	93 (33.9%)	137 (37.5%)	46 (24.2%)
**Sociodemographic** **variables**						
Sex						
Female	27.0 (14.6-41.7)	305 (54.9%)	141 (50.2%)	164 (59.9%)	192 (52.6%)	113 (59.5%)
Male	27.1 (14.6-39.6)	250 (45.1%)	140 (49.8%)	110 (40.1%)	173 (47.4%)	77 (40.5%)
Age group (years)						
40–49	33.3 (25.0-45.8)	73 (13.2%)	24 (8.5%)	49 (17.9%)	50 (13.0%)	23 (12.1%)
50–59	27.1 (14.6-41.7)	146 (26.3%)	62 (22.1%)	84 (30.7%)	90 (24.7%)	56 (29.5%)
60–69	27.1 (18.8-41.7)	186 (33.5%)	99 (35.2%)	87 (31.7%)	127 (34.8%)	59 (31.0%)
70 or older	22.9 (10.4-35.4)	150 (27.0%)	96 (34.2%)	54 (19.7%)	98 (26.9%)	52 (27.4%)
Age (years), mean (SD)	--	62.4 (10.5)	64.5 (10.3)	60.3 (10.3)	62.2 (10.3)	62.9 (10.9)
Self-identified ethnicity						
Mixed	27.1 (16.7-41.7)	438 (78.9%)	167 (59.4%)	271 (98.9%)	287 (78.6%)	151 (79.5%)
White	25.0 (12.5-37.5)	33 (5.9%)	31 (11.0%)	2 (0.7%)	18 (4.9%)	15 (7.9%)
Quechua	27.1 (17.7-48.4)	30 (5.4%)	30 (10.7%)	0 (0%)	22 (6.0%)	8 (4.2%)
Black	27.1 (8.3-39.6)	17 (3.1%)	17 (6.0%)	0 (0%)	12 (3.3%)	5 (2.6%)
Aymara	11.5 (6.8-16.1)	2 (0.4%)	2 (0.7%)	0 (0%)	1 (0.3%)	1 (0.5%)
Indigenous	35.4 (35.4-35.4)	1 (0.2%)	0 (0%)	1 (0.4%)	1 (0.3%)	0 (0%)
Other	18.8 (10.9-35.9)	6 (1.1%)	6 (2.1%)	0 (0%)	4 (1.1%)	2 (1.1%)
Unknown	20.8 (4.2-38.5)	28 (5.0%)	28 (10.0%)	0 (0%)	20 (5.5%)	8 (4.2%)
Marital status						
Single	25.0 (11.5-42.7)	160 (28.8%)	101 (35.9%)	59 (21.5%)	110 (30.1%)	50 (26.3%)
With a partner	27.1 (16.7-41.7)	395 (71.2%)	180 (64.1%)	215 (78.5%)	255 (69.9%)	140 (73.7%)
Presence of caregiver						
No	27.1 (14.6-41.7)	479 (86.3%)	241 (85.8%)	238 (86.9%)	309 (84.7%)	170 (89.5%)
Yes	27.1 (12.5-42.7)	76 (13.7%)	40 (14.2%)	36 (13.1%)	56 (15.3%)	20 (10.5%)
Education level						
Primary or less	29.2 (18.8-43.8)	128 (23.1%)	41 (14.6%)	87 (31.8%)	79 (21.6%)	49 (25.8%)
High school	28.1 (18.8-41.7)	248 (44.7%)	120 (42.7%)	128 (46.7%)	172 (47.1%)	76 (40.0%)
Superior	22.9 (10.4-37.5)	179 (32.3%)	120 (42.7%)	59 (21.5%)	114 (31.2%)	65 (34.2%)
Socioeconomic status						
Lower	29.2 (16.7-45.8)	124 (21.6%)	68 (23.1%)	56 (20.1%)	92 (24.5%)	32 (16.2%)
Lower-middle	29.1 (22.9-41.7)	106 (18.5%)	50 (17.0%)	56 (20.1%)	76 (20.2%)	30 (15.2%)
Middle	27.1 (14.6-39.6)	126 (22.0%)	67 (22.7%)	59 (21.2%)	78 (20.7%)	48 (24.2%)
Upper-middle	22.9 (12.5-35.4)	105 (18.3%)	52 (17.6%)	53 (19.0%)	64 (17.0%)	41 (20.7%)
Upper	27.1 (12.5-43.8)	113 (19.7%)	58 (19.7%)	55 (19.7%)	66 (17.6%)	47 (23.7%)
**Clinical variables**						
Number of chronic conditions, median (IQR)	--	2 (2–3)	3 (2–3)	2 (2–2)	2 (2–3)	2 (2–3)
Number of daily medications, mean (SD)	--	3.2 (1.7)	4.0 (2.0)	2.5 (1.0)	3.2 (1.7)	3.4 (1.7)
Insulin use						
No	27.1 (16.7-39.6)	446 (80.4%)	187 (66.6%)	259 (94.5%)	280 (76.7%)	166 (87.4%)
Yes	22.9 (10.4-43.8)	109 (19.6%)	94 (33.5%)	15 (5.4%)	85 (23.3%)	24 (12.6%)
Depressive symptoms – PHQ-8, median (IQR)	--	3 (0-7)	4 (2–8)	1 (0-5)	3 (0-7)	2 (0-6)
Moderate-Severe depressive symptoms						
No (PHQ-8 < 10 points)	25.0 (12.5-37.5)	482 (86.9%)	221 (78.7%)	261 (95.3%)	310 (84.9%)	172 (90.5%)
Yes (PHQ-8 ≥ 10 points)	37.5 (22.9-50.0)	73 (13.1%)	60 (21.3%)	13 (4.7%)	55 (15.1%)	18 (9.5%)
Wheelchair use						
No	27.1 (14.6-41.7)	515 (92.8%)	250 (88.9%)	265 (96.7%)	330 (90.4%)	185 (97.4%)
Yes	22.9 (11.5-36.5)	40 (7.2%)	31 (11.0%)	9 (3.3%)	35 (9.6%)	5 (2.6%)
Outpatient consultations in the last year, median (IQR)	--	10 (4–12)	6 (4–12)	11 (4–12)	10 (4–12)	10 (4–12)
Hospitalizations in the last year, median (IQR)	--	0	0	0	0	0
**Social, health and healthcare indicators**						
Family & social support – Gijon scale, median (IQR)	–	4 (4–6)	5 (4–7)	4 (3–4)	5 (4–6)	4 (3–5)
Social risk – Gijon scale categories						
Low (Gijon ≤7 points)	27.1 (14.6-39.6)	490 (88.3%)	229 (81.5%)	261 (95.3%)	311 (81.2%)	179 (94.2%)
Intermediate (Gijon 8–9 points)	31.3 (18.8-43.8)	47 (8.5%)	40 (14.2%)	7 (2.6%)	38 (10.4%)	9 (4.7%)
High (Gijon ≥10 points)	34.4 (22.9-50.0)	18 (3.2%)	12 (4.3%)	6 (2.2%)	16 (4.4%)	2 (1.1%)
Quality of life domains – EQ-5D-3L						
Mobility (some/extreme problem)	25.0 (12.5-39.6)	192 (34.6%)	141 (50.2%)	51 (18.6%)	142 (38.9%)	50 (26.3%)
Self-care (some/extreme problem)	27.1 (14.6-43.8)	61 (10.9%)	38 (13.5%)	23 (8.4%)	51 (13.9%)	10 (5.3%)
Usual activities (some/extreme problem)	27.1 (14.6-45.8)	110 (19.8%)	72 (25.6%)	38 (13.9%)	87 (23.8%)	23 (12.1%)
Pain/discomfort (some/extreme problem)	45.8 (18.8-45.8)	222 (40.0%)	142 (50.5%)	80 (29.2%)	160 (43.8%)	62 (32.6%)
Anxiety/depression (some/extreme problem)	33.3 (20.8-48.9)	116 (20.9%)	87 (30.9%)	29 (10.6%)	87 (23.8%)	29 (15.3%)
Quality of Life, median (IQR)	--	80 (60-90)	73 (50-80)	80 (70-90)	80 (60-90)	80 (70-90)
Medication adherence – ARMS, median (IQR)	--	17 (15–19)	16 (14–19)	17 (15–20)	17 (15–19)	17 (14–20)
Medication adherence, categories						
Poor (ARMS >12 points)	27.1 (6.3-37.5)	514 (92.6%)	243 (86.5%)	271 (98.9%)	344 (94.2%)	170 (89.5%)
Good (ARMS ≤12 points)	20.8 (16.7-41.7)	41 (7.4%)	38 (13.5%)	3 (1.1%)	21 (5.7%)	20 (10.5%)
Patient Assessment of Chronic Illness Care, PACIC, mean (SD)	--	2.8 (1.0)	2.2 (0.8)	3.4 (0.8)	2.7 (1.0)	3.1 (0.9)

*Data are presented as n (%) unless otherwise specified. Continuous variables are presented as mean (SD) or median (IQR), as indicated.

### Overview of chronic conditions and health indicators of the study sample

One-fifth of participants used insulin, with lower rates in Tumbes (5%) than Lima (34%) and in private (13%) than public services (23%). The average number of daily medications was 3, and only 7% reported wheelchair use, though this was more common in Lima and among those using public services. Mental health disparities were observed, with moderate to severe depressive symptoms more common in Lima than Tumbes (20% vs. 5%) and in public compared to private services (15% vs. 10%).

The median number of chronic conditions was 2 (IQR 2–3), with a median of 10 outpatient consultations (IQR 4–12) and no hospitalizations in the past year. Quality of life scores on the EQ-5D-3L visual analog scale had a median of 80 (IQR 60–90), reflecting relatively high self-rated quality of life, though 50% of participants in Lima reported some to extreme mobility problems (compared to 19% in Tumbes), and issues with usual activities were 10% higher among those in Lima and using public services. Overall, 20% and 40% of participants reported some to extreme problems with anxiety and pain, respectively.

Medication adherence scores (ARMS) had a median of 17, with good adherence (≤12 points) seen in 7.4% of the sample. Most participants (88%) had low social risk per the Gijon scale; however, high social risk (Gijon scores ≥10) was twice as prevalent in Lima compared to Tumbes (4% vs. 2%) and four times higher in public than private facilities. Participants in private facilities reported a slightly better perception of alignment of their treatment to the chronic care model as indicated by PACIC scores, compared to those in public facilities, with scores also higher among participants in semi-urban Tumbes compared to those in highly urban Lima.

### Patterns of treatment burden in the study population

In the overall sample, the median MTBQ score was 27.1, and distributions by exposures are shown in Fig 3. Higher MTBQ scores were reported by participants with some to extreme pain/discomfort on the EQ-5D-3L scale and those with moderate to severe depressive symptoms, with median scores of 45.8 and 37.5, respectively. Participants aged 40–49 years and those with anxiety/depression also had higher median MTBQ scores of 33.3. In contrast, patients aged 70 or older, those with higher education, and those with upper-middle socioeconomic status reported the lowest MTBQ scores, each with a median of 22.9. Additionally, there was a gradient of higher MTBQ scores corresponding to increasing categories of social risk on the Gijon scale, from low to intermediate to high social risk.

**Fig 3:**
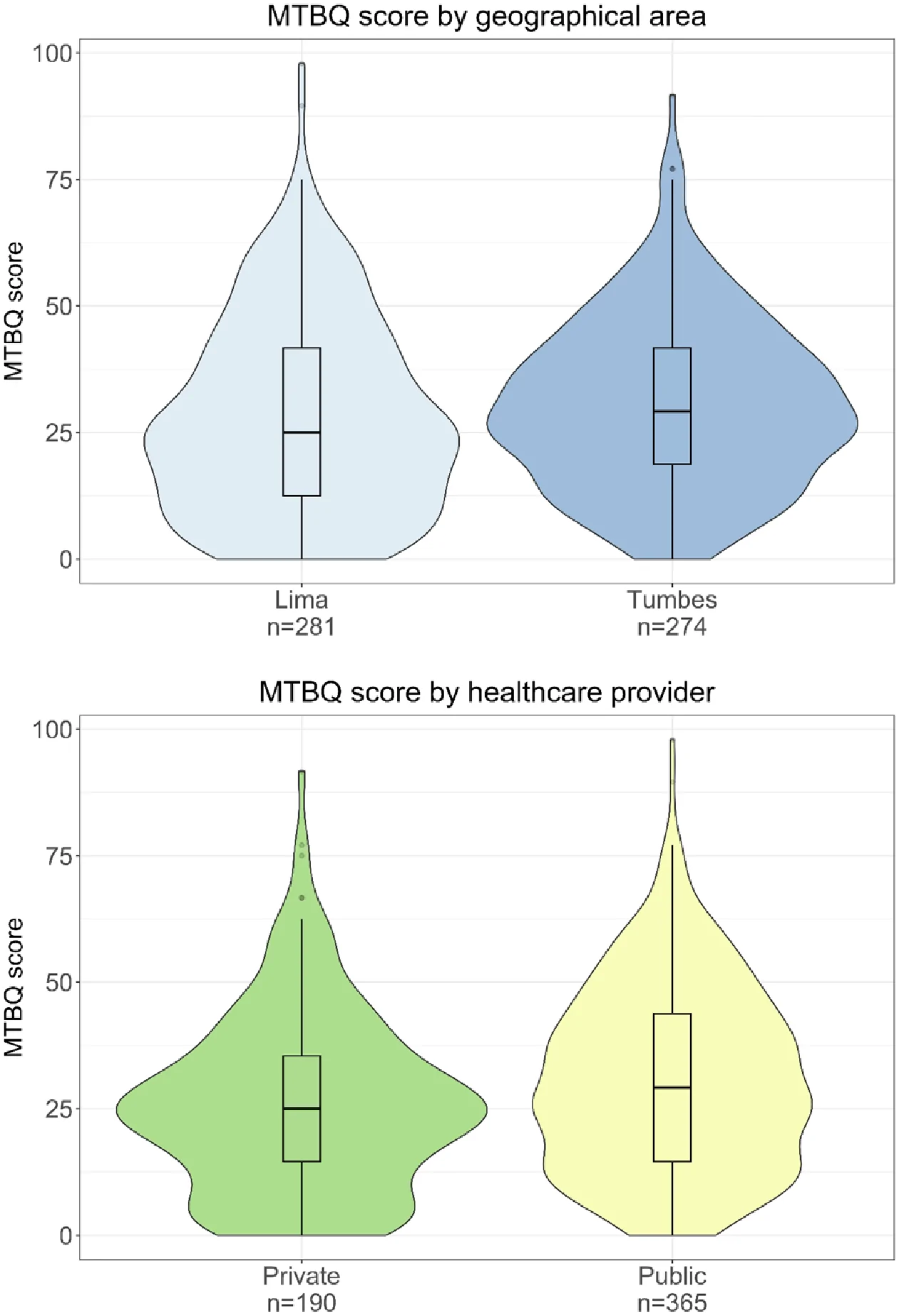
MTBQ scores by setting and healthcare provider.

An itemized distribution of MTBQ responses is presented in Table F in S1 Appendix Among reported challenges, 32% of participants found scheduling or obtaining appointments with healthcare providers extremely or very difficult, 28% reported difficulty accessing healthcare services during evenings or weekends, 26% experienced inconsistent care from different professionals at each visit, and 18% found paying for medications or medical equipment challenging.

### Association between geographical area and healthcare provider and treatment burden

Results of the models for differences in MTBQ median scores are shown in Table 3. Treatment burden was slightly higher in semi-urban Tumbes than in urban Lima and in public vs. private facilities.

**Table 3:** Median differences in MTBQ scores by geographical area and healthcare provider.

		Total MTBQ Standardized Score							
	N	Median (IQR)	Mean (SD)	Unadjusted model		Capacity-adjusted model		Workload-adjusted model	
				β	95% CI	β	95% CI	β	95% CI
Total sample	555	27.1 (14.6-41.7)	29.2 (18.1)						
**Median differences (Quantile** **regression)**									
Geographical area									
Lima, urban	281	25.0 (12.5-41.7)		Ref.		Ref.		Ref.	
Tumbes, semi-urban	274	29.2 (18.8-41.7)		4.2	0.5 to 7.8	2.1	-1.9 to 6.1	6.1	2.9 to 9.3
Healthcare provider									
Public	365	29.2 (14.6-43.8)		Ref.		Ref.		Ref.	
Private	190	25.0 (14.6-35.4)		-4.2	-7.5 to -0.8	-6.3	-9.8 to -2.7	-6.6	-9.3 to -3.9

95% CI = 95% confidence interval; IQR = Interquartile range, estimates with p-values <0.05 are shown in bold. Unadjusted model: Regression models were fitted using the MTBQ score as the dependent variable, and robust standard errors. Capacity-adjusted model: adjusted by sociodemographic variables (sex, age group, marital status, education level, and socioeconomic status) Workload-adjusted model: capacity-adjusted model plus clinical variables (caregiver, insulin use, wheelchair use, number of consultations, number of hospitalizations, number of medications, Patient Health Questionnaire-8).

In terms of geographical differences, the unadjusted model indicated that patients in Tumbes had a median MTBQ score 4.2 points higher than those in Lima. However, in the workload-adjusted model there was a strong association between study site and treatment burden, with patients in semi-urban Tumbes having, on average, 6.1 points higher MTBQ scores than those in urban Lima.

For healthcare provider differences, the unadjusted model indicated that patients in private facilities had a median MTBQ score 4.2 points lower than those in public facilities. Further adjustments strengthened this association, with the fully adjusted models showing a 6.6-point lower treatment burden score in private facilities.

Text D in S1 Appendix shows a summary of the findings alongside the conceptual model and theories underpinning treatment burden.

In a post-hoc analysis (Table 4), adjustment for depression (PHQ-8 score) explained the change in significance between the capacity-adjusted and workload-adjusted models. The difference in MTBQ scores between Tumbes and Lima was not significant in the capacity-adjusted model (β = 2.1; 95% CI: −1.9 to 6.1) but became significant after adjustment for depression (β = 6.9; 95% CI: 3.1 to 10.6), similar to the fully workload-adjusted model (β = 6.3; 95% CI: 2.6 to 10.1). Depression was associated with higher MTBQ scores in both settings (Table 5), with a stronger apparent association in Lima than in Tumbes. Consequently, adjustment for depression increased the estimated difference in MTBQ scores between geographical areas, suggesting that depressive symptoms may be an important confounder of the association between geographical area and treatment burden.

**Table 4:** Change in median differences in MTBQ scores by geographical area according to different variable adjustment.

	β (95% CI) Reference: Lima, urban Comparison group: Tumbes, semi-urban
Crude model	**4.2 (0.5 to 7.8)**
Depression only-adjusted model	**6.9 (3.3 to 10.7)**
Capacity-adjusted model (does not include depression)	2.1 (-1.9 to 6.1)
Capacity-adjusted model and depression	**6.9 (3.1 to 10.6)**
Workload-adjusted model (excluding depression)	2.2 (-1.6 to 6.0)
Workload-adjusted model (including depression)	**6.3 (2.6 to 10.1)**

Unadjusted model: Regression models were fitted using the MTBQ score as the dependent variable, and robust standard errors.

Capacity-adjusted model: adjusted by sociodemographic variables (sex, age group, marital status, education level, and socioeconomic status).

Workload-adjusted model: capacity-adjusted model plus clinical variables (caregiver, insulin use, wheelchair use, number of consultations, number of hospitalizations, number of medications, Patient Health Questionnaire-8).

**Table 5:** Median MTBQ scores according to geographical area and depression status.

Geographical area	Depression category	Unadjusted Median (IQR)
Lima, urban	Mild depression	22.9 (10.3-37.5)
	Moderate/Severe Depression	38.5 (22.9-48.9)
Tumbes, semi-urban	Mild depression	29.1 (18.8-39.5)
	Moderate/Severe Depression	37.5 (25.0-56.2)

## Discussion

### Key findings

This study provides novel insights into treatment burden among individuals with MLTC in Peru, highlighting differences across geographical locations and healthcare providers. Despite the high levels of MTBQ found in the study, the observed difference of around 6 points in MTBQ scores underscores a marked disparity, illustrating the pronounced impact of geography and healthcare provider type on patient experiences. These differences disproportionately disadvantage patients living with MLTC in rural settings and receiving care in public services, emphasizing the complex and demanding nature of managing MLTC and the extensive workload associated with seeking and adhering to care.

The theories of burden of treatment, patient capacity, and cumulative complexity contextualize our analytical approach [7,9,11,44]. While unadjusted models confirm differences between exposure groups, Lima-Tumbes and Public-Private, adjusted models afford deeper insights. Treatment burden is shaped by daily workload and individual capacity. The adjustment strategy used in our models attempts to cluster characteristics that can serve as proxies for capacity and workload. On one hand, socio-demographic variables tend to reflect the individual’s capacity and ability to manage both treatment burden and their everyday life. On the other hand, the clinical variables act as proxies for the effort required to manage an illness. Many of these factors not only contribute to the treatment burden but also represent elements of that burden themselves, which makes our model conservative yet reflective of the complexity inherent in the concept of treatment burden (Text D in S1 Appendix).

### Comparison with existing literature

In our study, treatment burden, as measured by the MTBQ, was substantially higher than what has been documented in previous research [40–42]. Existing metrics of treatment burden remain limited in capturing the unique context of LMICs, such as healthcare access barriers, reliance on social networks for care, and the influence of cultural factors. Median scores of MTBQ in other contexts fluctuate between 5.8 to 20.8 [42,45], much lower than our findings (median 27.1). Findings from Africa have highlighted similar contextual challenges, such as poverty, precarious healthcare infrastructure, limited access to medicines and services or even the “lack of” them, and the reliance on social networks for managing chronic conditions [46–51]. Originally developed in stable, high-resource environments, current metrics of treatment burden may fail to capture the reality of healthcare access barriers faced in these regions [46,49,52], needing to broaden the scope of treatment burden assessments to better reflect LMIC contexts.

### Relevance for healthcare delivery and public health

The COVID-19 pandemic exposed severe deficiencies [53], placing Peru and many Latin American countries among those with the highest mortality rates globally [54]. Systemic barriers rooted in poor primary healthcare organization or more proximal to the patient make access to care and care coordination difficult. Emerging evidence from China suggests that family doctor-led care, rather than specialist-driven care, is associated with lower treatment burden, offering a potential model for adaptation [55].

Treatment burden serves as an important indicator of how well healthcare systems are responding to the rise in MLTC and can act as a foundation for designing patient-centered interventions [56,57]. Treatment burden in the context of MLTC is complicated by the wide array of potential combinations of chronic conditions [8,58]. To improve comparability, we anchored MLTC with diabetes as an index condition, a tracer to benchmark health system performance [21,59].

### Strengths and limitations

The geographic variation we examined does not account for remote or hard-to-reach communities, nor does it encompass the full diversity of public-private healthcare providers. Consecutive sampling is practical and inclusive of eligible cases, but because it is non-random, it may introduce selection bias and limit generalisability. However, this approach was chosen to maximize coverage within the recruitment period, and we enrolled nearly all eligible patients attending the participating facilities during that time.

The field of treatment burden research is still evolving, and emerging research is exploring simpler and shorter tools to measure treatment burden that may be more suitable for LMIC settings, acknowledging the need for more contextually relevant measures of treatment burden in settings where healthcare access and resources may differ significantly from high-income countries [46,47,49]. Causality cannot be established and our findings are intended to inform future research and serve as a basis for hypothesis generation (Text D in S1 Appendix). In Peru, individuals with MLTC face a higher treatment burden in public and semi-urban healthcare settings compared to private and urban settings. These findings underscore the urgent need for targeted interventions, particularly in resource-limited areas. Strengthening primary care with a patient-centered focus through minimally disruptive care is essential to reduce disparities and alleviate treatment burden [8].

## Supporting information

S1 Appendix Supplmentary Text A–D and Tables A–F. **Text A.** Multimorbidity Treatment Burden Questionnaire (MTBQ), self-administered Spanish version. **Text B.** Multimorbidity Treatment Burden Questionnaire (MTBQ), Spanish version administered by the interviewer. **Text C.** Stata script used in the analysis. **Text D.** Summary of study findings alongside models, theories and contextual factors in the Peruvian setting. **Table A.** Evidence of validation of the Multimorbidity Treatment Burden Questionnaire (MTBQ). **Table B.** Definition and details of social, health and healthcare-related measures. **Table C.** Observed power estimations to assess the difference of the Multimorbidity Treatment Burden (MTBQ) score by geographical area and healthcare provider. **Table D.** Comparison of included and excluded participants. **Table E.** Description of chronic conditions of the study sample. **Table F.** Distribution of scores by item of the Multimorbidity Treatment Burden Questionnaire (MTBQ).
(DOCX)
