# Supplementary material for: Disparities in treatment burden in Peru among people with multiple long-term conditions by geography and healthcare facility: A cross-sectional study
Source: PLOS Glob Public Health. 2026 Sep 29;6(9):e0007355. doi: 10.1371/journal.pgph.0007355 (PMC13623086; doi:10.1371/journal.pgph.0007355)
Supplement: Supplementary file 1 [file pgph.0007355.s003.pdf]

29 June 2026

Dr. Mark G. Shrime
Editor, BMJ Global Health

Dear Dr. Shrime,

We are grateful to you and the reviewer for the time evaluating our manuscript. The comments have been very constructive and have helped us improve the work considerably. We have revised the manuscript thoroughly and provide below the details to each of the comments. All changes are highlighted with tracked-changes in the manuscript.

We hope that the revised manuscript meets the standards of PLOS Global Health and we look forward to your decision.

Edition comments:

1. **Comment 1 (Financial Disclosure):** Please amend your detailed Financial Disclosure statement. This is published with the article. It must therefore be completed in full sentences and contain the exact wording you wish to be published.

Response: Thank you for this guidance. We have revised the Financial Disclosure statement to include full sentences with all required information: funding sources with grant numbers, author initials linked to each grant, and an explicit statement confirming that the funders had no role in any aspect of the study. Please see the revised Funding section, lines 359–360.

2. **Comment 2 (Discussion section):** Your manuscript is missing the following sections: Discussion. Please ensure these are present, and in the correct order, and that any references to subheadings in your main text are correct.

Response: Thank you for this observation. We had inadvertently omitted a clearly labelled Discussion section. We have now added it in the correct position following the Results, in accordance with PLOS Global Health submission guidelines. Please see line 290.

3. **Comment 3 (Supporting Information legends):** We have noticed that you have uploaded Supporting Information files, but you have not included a list of legends. Please add a full list of legends for your Supporting Information files after the references list.

Response: We apologise for this omission. A complete list of legends for all Supporting Information files has been added after the references list, as required. Please see lines 566–583.

4. **Comment 4 (Data Availability Statement):** We note that your Data Availability Statement is currently as follows: "All relevant data are within the manuscript and its Supporting Information file." Please confirm at this time whether or not your submission contains all raw data required to replicate the results of your study. Authors must share the “minimal data set” for their submission

Response: Thank you for this clarification. We confirm that all data required to replicate the study findings are available in supplementary information S11. The dataset has been deposited in Figshare (DOI: 10.6084/m9.figshare.28385753) and the Data Availability Statement has been updated accordingly.

5. **Comment 5 (Copyright – Figure 1):** Some material included in your submission may be copyrighted. Figure 1: please (a) provide a direct link to the base layer of the

map (i.e., the country or region border shape) and ensure this is also included in the figure legend; and (b) provide a link to the terms of use / license information for the base layer image or shapefile. We cannot publish proprietary or copyrighted maps (e.g. Google Maps, Mapquest) and the terms of use for your map base layer must be compatible with our CC-BY 4.0 license.

Response: Thank you for raising this issue. We have updated the Figure 1 legend to include a direct link to the base layer map source (Wikimedia Commons) and confirmed that the license (CC BY-SA 4.0) is compatible with PLOS's CC BY 4.0 license. The attribution now reads: " Map created by the Communications Team at CRONICAS Center of Excellence in Chronic Diseases. Base layer adapted from: Peru Base Map, Wikimedia Commons (https://commons.wikimedia.org/wiki/File:Peru_Base_Map.png), licensed under CC BY-SA 4.0." An updated version of the figure has been submitted.

Reviewer Comments

1. **Comment 1 (Title):** Title needs improvement. Suggested title: "Disparities in treatment burden in Peru among people with multiple long-term conditions by geography and healthcare facility" or related.

Response: Thank you for this suggestion. We agree that the revised title better reflects the scope and focus of the study. We have adopted the suggested wording and added the study design descriptor. The title now reads: "Disparities in treatment burden in Peru among people with multiple long-term conditions by geography and healthcare facility: A cross-sectional study."

2. **Comment 2 (Background, line 52):** The background is insufficient to adequately describe the gap of the current study fill. Please add one strong paragraph to address this point using literature. In addition, please clarify further the relationships of the following: comorbidity, multimorbidity and multiple long-term conditions (MLTC).

Response: We thank the reviewer for this observation. We have strengthened the Background by adding a paragraph that explicitly articulates the evidence gap that the study addresses, supported by relevant literature (lines 67–73). We have also added a clarifying passage that explains the conceptual distinctions between comorbidity, multimorbidity, and MLTC, terms that are often used inconsistently in the literature (lines 57–59).

3. **Comment 3 (Method Study setting, line 77):** The study recruited sampling from private and public in two geographic regions-Lima and Tumbes. The sampling procedure is not clear. Did authors use simple random sampling, simple random sampling followed by systematic sampling, stratified sampling followed by simple random sampling? Please explain the sampling procedure in detail.

Response: We thank the reviewer for this observation. The manuscript lacked sufficient detail on the sampling procedure, which we have now clarified. Given the absence of comprehensive patient registries at the study sites, a common constraint in health research in LMICs, a consecutive sampling strategy was employed. This clarification has been added to the Methods section, Sample recruitment (line 114).

4. **Comment 4 (Sample recruitment, line 105):** For survey study, pilot test is essential for ensuring the validity and reliability of the study instruments. Did you conduct a pilot test? If so, please explain that. If not, please justify it.

Response: Thank you for this important comment. Prior to full-scale data collection, the MTBQ instrument underwent a rigorous cross-cultural adaptation process, which included forward and back-translation by bilingual researchers, cultural validation through focus groups conducted in two regions, and a final cognitive pilot test with 10

patients. Formal psychometric analyses of the instrument, including validity and reliability, are reported in a companion manuscript currently under peer review. In the Methods section, Study variables, has been revised to acknowledge this process in further detail (lines 141–143). Details of all other instruments used, which have undergone validation studies, are provided in Supplementary Table S5.

5. **Comment 5 (Study procedures, line 119 – Figure 2):** Please move Fig 2 under "Study procedures" so that it appears under this section for better flow and readability. Clarify the selection criteria in relation to geography (Lima vs Tumbes) and public vs private setting. What are the criteria for sampling of 326 and 282 from Lima and Tumbes? Please justify.

Response: Thank you for this suggestion. Figure 2 (Study flowchart) has been repositioned to the Study procedures section to improve narrative flow, with a cross-reference added at lines 135–136. Regarding the sample sizes: a larger number of individuals were contacted, 326 in Lima and 282 in Tumbes, because recruiting men from the private sector proved particularly challenging, and we sought to reduce the gender disparity in the final sample. After excluding participants with incomplete questionnaires, the final analytical sample comprised 281 participants in Lima and 274 in Tumbes.

6. **Comment 6 (Table 3, line 256):** Compared to Lima, Tumbes is insignificant in the capacity-adjusted model but significant on workload-adjusted model. Please clarify which variable specifically changes the significance level between the two models and provide an interpretation of this discrepancy.

Response: Thank you for this observation. To identify the factor responsible for the difference between models, we conducted additional post-hoc analyses by sequentially introducing variables from the workload-adjusted model. The results, now presented in Table 4, show that adjustment for depression (PHQ-8 score) explains the change in significance. The difference in MTBQ scores between Tumbes and Lima was not significant in the capacity-adjusted model ($\beta = 2.1$; 95% CI: −1.9 to 6.1) but became significant after adjustment for depression ($\beta = 6.9$; 95% CI: 3.1 to 10.6), similar to the fully workload-adjusted model ($\beta = 6.3$; 95% CI: 2.6 to 10.1). This finding has been interpreted in the text and its implications discussed, including the potential role of depressive symptoms as a confounder of the association between geographical area and treatment burden. Please see lines 277–289.

7. **Comment 7 (Conclusions):** The conclusions section currently contains Discussion points. Please create a separate Discussion section that interprets key findings, addresses how they compare with existing literature, and emphasises broader implications.

Response: We agree with the reviewer. We have now added clearly labelled Discussion section. Please see line 290.

8. **Comment 8 (References – Older citations):** Some references are quite old (e.g., from 2006), but overall the references are good.

Response: We acknowledge this observation. We have reviewed the references and retained older citations where they represent foundational or seminal work. We believe the overall reference list adequately reflects current evidence.

We thank the editorial team and reviewer once more for their thorough and constructive engagement with our manuscript. We believe the revised version addresses all the points raised and represents a substantially improved contribution.

Yours sincerely,

Silvana Perez Leon
CRONICAS Center of Excellence in Chronic Diseases
Universidad Peruana Cayetano Heredia
Av. Armendariz 497, Miraflores
Lima 18, Peru
+51 1 241 6978
silvanaplq@gmail.com
