# Supplementary material for: Disparities in treatment burden in Peru among people with multiple long-term conditions by geography and healthcare facility: A cross-sectional study
Source: PLOS Glob Public Health. 2026 Sep 29;6(9):e0007355. doi: 10.1371/journal.pgph.0007355 (PMC13623086; doi:10.1371/journal.pgph.0007355)
Supplement: Supplementary file 2 [file pgph.0007355.s001.docx]

**Supporting Information**

**S1 Appendix**

Supplement to: Perez-Leon S, Mendoza-Quispe D, Cuba-Fuentes MS, Bazo-Alvarez JC, Pesantes MA, Gaspar A, Zunt JR, Montori VM, Castillo JA, Carcausto E, Diaz N, Salirrosas K, Cornejo P, Miranda JJ.

Treatment burden in Peru across settings and regions among people with multiple long-term conditions: A cross-sectional study.

This appendix has been provided by the authors to give readers additional information about the work.

**Table of Contents**

[Text A: Multimorbidity Treatment Burden Questionnaire (MTBQ), self-administered Spanish version	3](#_Toc240107272)

[Text B. Multimorbidity Treatment Burden Questionnaire (MTBQ), Spanish version administered by the interviewer	4](#_Toc240107273)

[Table A. Evidence of validation of the Multimorbidity Treatment Burden Questionnaire (MTBQ)	5](#_Toc240107274)

[Table B. Definition and details of social, health and healthcare-related measures	6](#_Toc240107275)

[Table C. Observed power estimations to assess the difference of the Multimorbidity Treatment Burden (MTBQ) score by geographical area and healthcare provider	7](#_Toc240107276)

[Table D. Comparison of included and excluded participants	8](#_Toc240107277)

[Table E. Description of chronic conditions of the study sample	9](#_Toc240107278)

[Table F. Distribution of scores by item of the Multimorbidity Treatment Burden Questionnaire (MTBQ)	10](#_Toc240107279)

[Text C. Summary of study findings alongside models, theories and contextual factors in the Peruvian setting	11](#_Toc240107280)

[Text D. Stata script used in the analysis	14](#_Toc240107281)

[References for S1 Appendix	17](#_Toc240107282)

# Text A: Multimorbidity Treatment Burden Questionnaire (MTBQ), self-administered Spanish version

|  | Extremadamente difícil | Muy  difícil | Algo  difícil | Un poco difícil | No es  difícil | No aplica a mi caso |
| --- | --- | --- | --- | --- | --- | --- |
| Tomar muchas medicinas |  5 | 4 | 3 | 2 | 1 | 0 |
| Recordar cómo y cuándo tomar las medicinas |  5 | 4 | 3 | 2 | 1 | 0 |
| Pagar por medicinas con receta, medicinas de venta libre o equipos médicos |  5 | 4 | 3 | 2 | 1 | 0 |
| Recoger sus medicamentos con receta (con o sin pago) |  5 | 4 | 3 | 2 | 1 | 0 |
| Monitorear sus condiciones médicas (por ejemplo, chequear su presión arterial o azúcar en sangre, monitorear sus síntomas, etc.) |  5 | 4 | 3 | 2 | 1 | 0 |
| Programar o conseguir sus citas con profesionales de salud |  5 | 4 | 3 | 2 | 1 | 0 |
| Ser atendido por diferentes profesionales de salud en cada consulta |  5 | 4 | 3 | 2 | 1 | 0 |
| Asistir a sus citas con los profesionales de salud (por ejemplo, dejar de trabajar un tiempo, transportarse, etc.) |  5 | 4 | 3 | 2 | 1 | 0 |
| Conseguir atención de salud en las tardes/noches o los fines de semana |  5 | 4 | 3 | 2 | 1 | 0 |
| Obtener, de los profesionales de salud, información clara y actualizada de su condición médica |  5 | 4 | 3 | 2 | 1 | 0 |
| Seguir recomendaciones sobre cambios en su estilo de vida (ej. dieta y ejercicio, etc.) |  5 | 4 | 3 | 2 | 1 | 0 |
| Tener que contar con apoyo adicional de familiares y amigos (económico, emocional, tareas, etc.) |  5 | 4 | 3 | 2 | 1 | 0 |

# Text B. Multimorbidity Treatment Burden Questionnaire (MTBQ), Spanish version administered by the interviewer

| ***Leer las preguntas al entrevistado*** | Extremadamente difícil | Muy  difícil | Algo  difícil | Un poco difícil | No es  difícil | No aplica a mi caso |
| --- | --- | --- | --- | --- | --- | --- |
| Tomar muchas medicinas |  5 | 4 | 3 | 2 | 1 | 0 |
| Recordar cómo y cuándo tomar las medicinas |  5 | 4 | 3 | 2 | 1 | 0 |
| Pagar por medicinas con receta, medicinas de venta libre o equipos médicos |  5 | 4 | 3 | 2 | 1 | 0 |
| Recoger sus medicamentos con receta (con o sin pago) |  5 | 4 | 3 | 2 | 1 | 0 |
| Monitorear sus condiciones médicas (por ejemplo, chequear su presión arterial o azúcar en sangre, monitorear sus síntomas, etc.) |  5 | 4 | 3 | 2 | 1 | 0 |
| Programar o conseguir sus citas con profesionales de salud | 5 | 4 | 3 | 2 | 1 | 0 |
| Ser atendido por diferentes profesionales de salud en cada consulta | 5 | 4 | 3 | 2 | 1 | 0 |
| Asistir a sus citas con los profesionales de salud (por ejemplo, dejar de trabajar un tiempo, transportarse, etc.) | 5 | 4 | 3 | 2 | 1 | 0 |
| Conseguir atención de salud en las tardes/noches o los fines de semana | 5 | 4 | 3 | 2 | 1 | 0 |
| Obtener, de los profesionales de salud, información clara y actualizada de su condición médica | 5 | 4 | 3 | 2 | 1 | 0 |
| Seguir recomendaciones sobre cambios en su estilo de vida (ej. dieta y ejercicio, etc.) | 5 | 4 | 3 | 2 | 1 | 0 |
| Tener que contar con apoyo adicional de familiares y amigos (económico, emocional, tareas, etc.) | 5 | 4 | 3 | 2 | 1 | 0 |

# Table A. Evidence of validation of the Multimorbidity Treatment Burden Questionnaire (MTBQ)

| Year | Country | Language of the MTBQ | Reference |
| --- | --- | --- | --- |
| 2018 | UK | English | ^1^ |
| 2018 | UK | English | ^2^ |
| 2020 | UK | Chinese | ^3^ |
| 2021 | Canada | French | ^4^ |
| 2022 | Germany | German | ^5^ |
| 2022 | England | English | ^6^ |
| 2023 | Denmark | Danish | ^7^ |
| 2023 | Netherlands | Dutch | ^8^ |
| 2023 | Lithuania | Lithuanian | ^9^ |

# Table B. Definition and details of social, health and healthcare-related measures

| Questionnaire | Interpretation of Scores | References of validation studies |
| --- | --- | --- |
| **The Patient Health Questionnaire-8 (PHQ-8)**  Factor structure: unidimensional  Number of Items: 8  Score by item: 0 to 3 points | Total score: arithmetic sum of all items (range 0 to 24 points). Higher scores indicate higher depressive symptoms. A cut-off score of ≥10 was used to identify clinically significant depressive symptoms (no, yes). | ^10–12^ |
| **Patient Assessment of Chronic Illness Care (PACIC)**  Factor structure: unidimensional  Number of Items: 20  Score by item: 0 to 5 points | Total score: mean score of all items (range 1 to 5 points). Higher scores indicate better patient-reported chronic illness care. | ^13,14^ |
| **Adherence to Refill and Medication Scale (ARMS)**  Factor structure: unidimensional  Number of Items: 12  Score by item: 1 to 4 points | Total score: arithmetic sum of all items (range: 12-48 points). Higher scores indicate poor adherence.  Categorical score: Poor adherence (>12 points), Good adherence (12 or less points). | ^15^ |
| **Gijon Scale**  Factor structure: unidimensional  Number of Items: 3  Score by item: 1 to 5 points | Total score: arithmetic sum of all items (range 3 to 15 points). Higher scores indicate greater social risk.  Categorical score: low social risk (≥ 7 points), intermediate social risk (8-9 points) and high social risk (≥10 points). | ^16,17^ |
| **European Quality of Life - 5 Dimensions - Three‑Level (EQ-5D-3L)**  Factor structure:  Number of Items: 6  Score by item: Items 1 to 5 are each scored from 1 to 3; item 6 is a visual analog scale scored from 0 to 100 | Total score: arithmetic sum of all items (range 3 to 15 points). Higher scores indicate better quality of life. | ^18–20^ |

# Table C. Observed power estimations to assess the difference of the Multimorbidity Treatment Burden (MTBQ) score by geographical area and healthcare provider

| **Subgroup** | | | **Parameter *** | | | |
| --- | --- | --- | --- | --- | --- | --- |
|  |  |  | Sample size | Mean ** | Standard deviation | Power (%) |
| **Difference in MTBQ scores by geographical area** | | | | | | |
|  | Highly-urban Lima | | 281 | 27.9 | 19.3 | **Reference** |
|  |  | Semi-urban Tumbes (-2.5) | 274 | 25.4 | 16.6 | 37.3 |
|  |  | Semi-urban Tumbes (+2.5) |  | 30.4 |  | 37.3 |
|  |  | Semi-urban Tumbes (-5) |  | 22.9 |  | 90.5 |
|  |  | Semi-urban Tumbes (+5) |  | 32.9 |  | 90.5 |
|  |  | Semi-urban Tumbes (-10) |  | 17.9 |  | 100 |
|  |  | Semi-urban Tumbes (+10) |  | 37.9 |  | 100 |
|  | | | | | | |
| **Subgroup** | | | **Parameter *** | | | |
|  |  |  | Sample size | Mean ** | Standard deviation | Power (%) |
| **Difference in MTBQ scores by healthcare provider** | | | | | | |
|  | Public | | 365 | 30.9 | 18.2 | **Reference** |
|  |  | Private (-2.5) | 190 | 28.4 | 17.3 | 38.7 |
|  |  | Private (+2.5) |  | 33.4 |  | 38.7 |
|  |  | Private (-5) |  | 25.9 |  | 91.2 |
|  |  | Private (+5) |  | 35.9 |  | 91.2 |
|  |  | Private (-10) |  | 20.9 |  | 100 |
|  |  | Private (+10) |  | 40.9 |  | 100 |
| (*) Power was computed in Stata v16.1 using the command 'power twomeans' and considering the following parameters: the sample size and the standard deviation of the reference and comparison groups as observed in our study data. An alpha value of 0.05 was set.  (**) For the mean of the reference group, we used the mean observed in our study data. For the mean of the comparison groups, we tested different scenarios where their mean MTBQ score was above or below 2.5 points, 5 points, and 10 points compared to the reference group. | | | | | | |

Table D. Comparison of included and excluded participants

| Characteristics | | | STATUS | |
| --- | --- | --- | --- | --- |
|  |  |  | **Excluded** | **Included** |
|  |  |  | **N = 6 (%)** | **N = 555 (%)** |
| Sociodemographic variables | | |  |  |
|  | **Sex** | |  |  |
|  |  | Female | 1 (16.7%) | 305 (54.9%) |
|  |  | Male | 3 (50.0%) | 250 (45.1%) |
|  |  | Missing | 2 (33.3%) | - |
|  | **Age group (years)** | |  |  |
|  |  | 40 to 49 | - | 73 (13.2%) |
|  |  | 50 to 59 | - | 146 (26.3%) |
|  |  | 60 to 69 | 2 (33.3%) | 186 (33.5%) |
|  |  | 70 or older | 2 (33.3%) | 150 (27.0%) |
|  |  | Missing | 2 (33.3%) | - |
|  | **Age (years): mean (SD)** | | 66.5 (7.2) | 62.4 (10.5) |
|  | **Marital status** | |  |  |
|  |  | Single | 3 (50.0%) | 160 (28.8%) |
|  |  | With couple | 1 (16.7%) | 395 (71.2%) |
|  |  | Missing | 2 (33.3%) | - |
|  | **Education level** | |  |  |
|  |  | Primary or less | - | 128 (23.1%) |
|  |  | High-school | 1 (16.7%) | 248 (44.7%) |
|  |  | Superior | 3 (50.0%) | 179 (32.3%) |
|  |  | Missing | 2 (33.3%) | - |
|  | **Socioeconomic status** | |  |  |
|  |  | Lower | 5 (83.3%) | 111 (20.0%) |
|  |  | Lower-middle | - | 110 (19.8%) |
|  |  | Middle | 1 (16.7%) | 110 (19.8%) |
|  |  | Upper-middle | - | 146 (26.3%) |
|  |  | Upper | - | 78 (14.1%) |
| Clinical variables | | |  |  |
|  | **Presence of caregiver** | |  |  |
|  |  | No | 3 (50.0%) | 479 (86.3%) |
|  |  | Yes | 1 (16.7%) | 76 (13.7%) |
|  |  | Missing | 2 (33.3%) | - |
|  | **Insulin use** | |  |  |
|  |  | No | 2 (33.3%) | 446 (80.4%) |
|  |  | Yes | - | 109 (19.6%) |
|  |  | Missing | 4 (66.7%) | - |
|  | **Wheelchair use** | |  |  |
|  |  | No | 1 (16.7%) | 515 (92.8%) |
|  |  | Yes | 1 (16.7%) | 40 (7.2%) |
|  |  | Missing | 4 (66.7%) | - |
|  | **Number of chronic conditions: median (IQR)** | | 2.5 (2-4) | 2 (2-3) |
|  | **Outpatient consultations in the last year: median (IQR)** | | 2 (2-2) | 10 (4-12) |
|  | **Hospitalizations the last year: median (IQR)** | | - | 0 (0-0) |
| Healthcare instruments | | |  |  |
|  |  | Complete | - | 555 (100%) |
|  |  | Missing | 6 (100.0%) | - |

Abbreviations: SD = Standard deviation; IQR = Interquartile range

# Table E. Description of chronic conditions of the study sample

| Characteristics | | TOTAL SAMPLE |
| --- | --- | --- |
|  |  | **N = 555 (%)** |
| Diabetes, Yes | | 555 (100.0%) |
| Hypertension, Yes | | 383 (69.0%) |
| Cardiovascular disease, Yes | | 30 (5.4%) |
| Cerebrovascular disease, Yes | | 5 (0.9%) |
| Tuberculosis, Yes | | 4 (0.7%) |
| Asthma, Yes | | 15 (2.7%) |
| Chronic bronchitis, Yes | | 4 (0.7%) |
| Chronic Obstructive Pulmonary disease, Yes | | 1 (0.2%) |
| Ischemic Heart Disease, Yes | | 12 (2.2%) |
| Depression, Yes | | 19 (3.4%) |
| Other Mental Health Disorder, Yes | | 3 (0.5%) |
| Cancer, Yes | | 8 (1.4%) |
| Other disease, Yes | | 299 (53.9%) |
|  | Other: Endocrine disease | 71 / 299 (23.8%) |
|  | Other: Cardiovascular disease | 43 / 299 (14.4%) |
|  | Other: Gastrointestinal disease | 40 / 299 (13.4%) |
|  | Other: Kidney disease | 30 / 299 (10.0%) |
|  | Other: Osteomuscular disease | 22 / 299 (7.4%) |
|  | Other: Eye disease | 13 / 299 (4.4%) |
|  | Other: Respiratory disease | 3 / 299 (1.0%) |
|  | Other: Ear disease | 3 / 299 (1.0%) |
|  | Other: Skin disease | 3 / 299 (1.0%) |
|  | Other: Neurological disease | 2 / 299 (0.7%) |
|  | Other: Hematological disease | 2 / 299 (0.7%) |
|  | Other: Gynecological disease | 1 / 299 (0.3%) |
|  | Other: Infectious disease | 1 / 299 (0.3%) |
|  | Other: Two other diseases | 45 / 299 (15.0%) |
|  | Other: Three other diseases | 18 / 299 (6.0%) |
|  | Other: Five other diseases | 1 / 299(0.3%) |
|  | Other: Seven other diseases | 1 / 299 (0.3%) |

# Table F. Distribution of scores by item of the Multimorbidity Treatment Burden Questionnaire (MTBQ)

| Item | 4: Extremely difficult | 3: Very difficult | 2: Some difficult | 1: Little difficult | 0: Not difficult | 0: Does not apply |
| --- | --- | --- | --- | --- | --- | --- |
|  |  |  |  |  |  |  |
| 1. Tomar muchas medicinas. | 16 (2.9%) | 44 (7.9%) | 99 (17.8%) | 167 (30.1%) | 216 (38.9%) | 13 (2.3%) |
| 2. Recordar cómo y cuándo tomar las medicinas. | 6 (1%) | 33 (6%) | 99 (17.8%) | 165 (29.7%) | 242 (43.6%) | 10 (1.8%) |
| 3. Pagar por medicinas con recetas, medicinas de venta libre o equipos médicos. | 25 (4.5%) | 76 (13.7%) | 137 (24.7%) | 136 (24.5%) | 143 (25.8%) | 38 (6.9%) |
| 4. Recoger sus medicamentos con receta (con o sin pago). | 14 (2.5%) | 44 (7.9%) | 86 (15.5%) | 118 (21.3%) | 268 (48.3%) | 25 (4.5%) |
| 5. Monitorear sus condiciones médicas ( por ejemplo, chequear su presión arterial o azúcar en sangre, monitorear sus signos, etc). | 19 (3.4%) | 69 (12.4%) | 143 (25.8%) | 99 (17.8%) | 221 (39.8%) | 4 (0.7%) |
| 6: Programar o conseguir sus citas con profesionales de salud. | 69 (12.4%) | 107 (19.3%) | 114 (20.5%) | 108 (19.5%) | 147 (26.5%) | 10 (1.8%) |
| 7. Ser atendido por diferentes profesionales de salud en cada consulta. | 52 (9.3%) | 92 (16.6%) | 97 (17.5%) | 121 (21.8%) | 167 (30.1%) | 26 (4.7%) |
| 8. Asistir a sus citas con los profesionales de salud (por ejemplo, dejar de trabajar un tiempo, transportarse,etc). | 23 (4.1%) | 61 (11%) | 100 (18%) | 135 (24.3%) | 211 (38%) | 25 (4.5%) |
| 9. Conseguir atención de salud en las tardes/noches o los fines de semana. | 52 (9.4%) | 104 (18.7%) | 101 (18%) | 131 (23.6%) | 132 (23.8%) | 35 (6.3%) |
| 10. Obtener, de los profesionales de salud, información clara y actualizada de su condición médica. | 18 (3.2%) | 49 (8.8%) | 68 (12.3%) | 107 (19.3%) | 305 (55%) | 8 (1.4%) |
| 11. Seguir recomendaciones sobre cambios en su estilo de vida (ej. Dieta y ejercicios,etc). | 16 (2.9%) | 73 (13.2%) | 130 (23.4%) | 145 (26.1%) | 184 (33.2%) | 7 (1.3%) |
| 12. Tener que contar con apoyo adicional de familiares y amigos (económico, emocional,tareas,ect). | 12 (2.2%) | 39 (7%) | 91 (16.4%) | 161 (29%) | 220 (39.6%) | 32 (5.8) |
| 13. Pagar por otros gastos de salud, como consultas médicas, exámenes de laboratorio, terapia, etc. | 29 (5.2%) | 103 (18.6%) | 141 (25.4%) | 132 (23.8%) | 120 (21.6%) | 30 (5.4%) |

# Text C. Summary of study findings alongside models, theories and contextual factors in the Peruvian setting

| **Key concepts, models and theories, informing the burden of treatment** | |
| --- | --- |
| **Cumulative Complexity Model** emphasizes the interplay between a patient's workload (demands of managing health and daily life) and capacity (resources and abilities to manage that workload). It acknowledges that complexity accumulates over time and impacts access to and utilization of healthcare, as well as self-care and overall health. ^21^  **Patient Capacity Theory** is not simply about the resources a patient has, but their dynamic ability to mobilize resources to meet the demands of healthcare and daily life. Capacity is influenced by multiple factors, including Biography: how a person reshapes their life in the context of a chronic condition, Resources: the material and social resources available; Environment: the kindness, empathy, and feasibility of a treatment plan within their life; Work: The ability to manage both "patient work" (managing the illness) and "life work" (employment, family responsibilities); and, Social Networks: The support system available to the patient. ^22^  **Normalization Process Theory** helps us understand how new practices, including treatment regimens, become integrated into patients' daily routines. For treatment to be successfully adopted, it needs to be coherent (make sense to the patient), involve collective action (implementation), enable cognitive participation (engagement with others), and support reflexive monitoring (appraisal). ^23^  **Burden of Treatment Theory** highlights the subjective experience of treatment burden and refers to the overall workload that patients experience as they manage their health conditions. It includes the effort, tasks, and impact on daily life that arise from medical treatments and self-care demands. ^24^ | |
| **Lima, urban**  **Lower treatment burden** | **Tumbes, semi-urban**  **Higher treatment burden** |
| Treatment burden is a complex concept with multiple facets, and conceptual models and theories surrounding this concept can help generate hypotheses to explain the observed data. At first glance, the data might suggest that Lima (urban) would have a higher treatment burden—patients have a greater number of daily medications, higher insulin use (which could increase treatment workload), more frequent wheelchair use (indicating greater physical limitations), more moderate to severe depressive symptoms, lower quality of life and more social risk. However, the findings indicate that the treatment burden is actually higher in Tumbes (semi-urban).  What can the conceptual models and theories tell us? | |
| **Patient Capacity Theory** suggests that treatment burden is not only determined by medical demands but also by a patient's ability to manage them. In Tumbes, lower education levels, socioeconomic constraints, and competing responsibilities (such as work and caregiving) may reduce patients’ capacity to effectively integrate treatment into their daily lives, increasing their perceived burden. | |
| **Cumulative Complexity Model** highlights that treatment burden is compounded by additional life demands. The population in Tumbes is generally younger, meaning they have a greater overall workload due to professional responsibilities, caregiving, and other daily demands. This increased **general workload** creates an imbalance between the workload imposed by treatment and the patient’s capacity to manage it, leading to a disproportionate treatment burden. Additionally, patients in Tumbes may face structural barriers such as limited healthcare access, longer waiting times, and resource scarcity, further adding to the complexity of managing their conditions. Even if they take fewer medications, the effort required to obtain and adhere to treatment could be significantly higher. | |
| **Normalization Process Theory** emphasizes how patients incorporate treatment into their routines. In resource-limited settings like Tumbes, inconsistent access to medications and healthcare services may disrupt this process, leading to a greater struggle to maintain stable treatment routines, thereby increasing burden. | |
| **Burden of Treatment Theory** explains that the effort required to engage with healthcare—such as navigating an inefficient system, self-managing chronic conditions, and compensating for gaps in care—contributes to overall burden. In Tumbes, limited healthcare infrastructure and financial constraints may mean that patients must exert more effort to receive adequate care, intensifying their treatment burden despite having fewer prescribed interventions. While patients in Tumbes appear to have a stronger social support system, it does not seem sufficient to offset the limitations that contribute to their increased burden. | |
| **Public healthcare provider**  **Higher treatment burden** | **Private healthcare provider**  **Lower treatment burden** |
| The differences between public and private healthcare can be analyzed to understand their impact on treatment burden. Key factors such as education, socioeconomic status, and access to medical resources shape a patient’s ability to manage their treatment effectively. The following the conceptual models and theories provide insight into these disparities: | |
| **Patient Capacity Theory** helps explain disparities between public and private healthcare providers through three of its five key elements. Resources: Private healthcare patients generally have higher education and financial resources, enhancing their ability to understand medical instructions and navigate the system, thereby reducing their treatment burden. Environment: Private healthcare settings are less complex, more patient-centered, and less crowded, which improves patient capacity and eases treatment management. Work: A stronger perception of treatment success and better medication adherence in private settings may reinforce engagement and self-care, further lowering the burden of treatment. | |
| **Cumulative Complexity Model** explains that treatment burden is not just about the medical regimen itself but also about the overall workload patients must manage. Private healthcare patients generally have more financial resources and better access to healthcare services, which can reduce complexity by minimizing barriers such as long waiting times, treatment interruptions, and financial constraints. In contrast, public healthcare patients may need to invest additional effort to secure medical care, making treatment management more challenging. | |
| **Burden of Treatment Theory** highlights the importance of relational networks and social capital in managing treatment burden. People in private healthcare settings appear to have lower social risk and greater access to services, which can facilitate treatment management. Additionally, private healthcare patients often have the necessary resources to comply with treatment regimens, while public healthcare patients may experience interruptions in care due to financial and systemic limitations, ultimately increasing their overall treatment burden. | |

# Text D. Stata script used in the analysis

*Description by geographic variables (lima vs. tumbes)

**************************

tab mtbq_tertiles_stand_score departam, m col nokey

tab sex1 departam, m col nokey

tab agecat1 departam, m col nokey

bysort departam: summarize age3,d

tab ethnicity departam, m col nokey rowsort

tab est_civil1 departam, m col nokey

tab caregiver1 departam, m col nokey

tab education1 departam, m col nokey

tab quintiles_assets departam, m col nokey

bysort departam: summarize num_conditions_dbplus,d

bysort departam: summarize num_med_total,d

tab insulin_use departam,m col nokey

bysort departam: summarize phq8_total_score,d

tab ph8_depression_modsev departam,m col nokey

tab wheelchair_use departam,m col nokey

bysort departam: summarize consultations_lastyear,d

bysort departam: summarize hosp_lastyear,d

bysort departam: summarize gijon_total_score,d

tab gijon_social_3cats departam,m col nokey

tab eq_cat01 departam,m col nokey

tab eq_cat02 departam,m col nokey

tab eq_cat03 departam,m col nokey

tab eq_cat04 departam,m col nokey

tab eq_cat05 departam,m col nokey

bysort departam: summarize eq_06,d

bysort departam: summarize arms_total_score,d

tab arms_cat departam,m col nokey

bysort departam: summarize pacic_mean_score,d

*Description by healthcare provider

**************************

tab mtbq_tertiles_stand_score estable_cat, m col nokey

tab sex1 estable_cat, m col nokey

tab agecat1 estable_cat, m col nokey

bysort estable_cat: summarize age3,d

tab ethnicity estable_cat, m col nokey rowsort

tab est_civil1 estable_cat, m col nokey

tab caregiver1 estable_cat, m col nokey

tab education1 estable_cat, m col nokey

tab quintiles_assets estable_cat, m col nokey

bysort estable_cat: summarize num_conditions_dbplus,d

bysort estable_cat: summarize num_med_total,d

tab insulin_use estable_cat,m col nokey

bysort estable_cat: summarize phq8_total_score,d

tab ph8_depression_modsev estable_cat,m col nokey

tab wheelchair_use estable_cat,m col nokey

bysort estable_cat: summarize consultations_lastyear,d

bysort estable_cat: summarize hosp_lastyear,d

bysort estable_cat: summarize gijon_total_score,d

tab gijon_social_3cats estable_cat,m col nokey

tab eq_cat01 estable_cat,m col nokey

tab eq_cat02 estable_cat,m col nokey

tab eq_cat03 estable_cat,m col nokey

tab eq_cat04 estable_cat,m col nokey

tab eq_cat05 estable_cat,m col nokey

bysort estable_cat: summarize eq_06,d

bysort estable_cat: summarize arms_total_score,d

tab arms_cat estable_cat,m col nokey

bysort estable_cat: summarize pacic_mean_score,d

**TABLE: BIVARIATE ANALYSIS

*----------------------------

bysort mtbq_tertiles_stand_score: summarize mtbq_tot_stand_score,d

bysort sex1: summarize mtbq_tot_stand_score,d

bysort agecat1: summarize mtbq_tot_stand_score,d

bysort ethnicity: summarize mtbq_tot_stand_score,d

bysort est_civil1: summarize mtbq_tot_stand_score,d

bysort caregiver1: summarize mtbq_tot_stand_score,d

bysort education1: summarize mtbq_tot_stand_score,d

bysort quintiles_assets: summarize mtbq_tot_stand_score,d

bysort insulin_use: summarize mtbq_tot_stand_score,d

bysort ph8_depression_modsev: summarize mtbq_tot_stand_score,d

bysort wheelchair_use: summarize mtbq_tot_stand_score,d

bysort gijon_social_3cats: summarize mtbq_tot_stand_score,d

bysort eq_cat01: summarize mtbq_tot_stand_score,d

bysort eq_cat02: summarize mtbq_tot_stand_score,d

bysort eq_cat03: summarize mtbq_tot_stand_score,d

bysort eq_cat04: summarize mtbq_tot_stand_score,d

bysort eq_cat05: summarize mtbq_tot_stand_score,d

bysort arms_cat: summarize mtbq_tot_stand_score,d

**TABLE: MULTIPLE REGRESSION ANALYSIS

*MEDIAN / QUANTILE REGRESSION

*crude:

qreg mtbq_tot_stand_score i.departam, vce(robust)

qreg mtbq_tot_stand_score i.estable_cat, vce(robust)

*Capacity-adjusted model: adjusted by sociodemographic variables (sex, age group, marital status, education level, and socioeconomic status)

qreg mtbq_tot_stand_score i.departam i.estable_cat i.sex1 i.agecat1 i.est_civil1 i.education1 i.quintiles_assets, vce(robust)

vif, uncentered

*Workload-adjusted model: capacity-adjusted model plus clinical variables (caregiver, insulin use, wheelchair use, number of consultations, number	hospitalizations, number of medications, Patient Health Questionnaire-8).

qreg mtbq_tot_stand_score i.departam i.estable_cat i.sex1 i.agecat1 i.est_civil1 i.education1 i.quintiles_assets ///

i.caregiver1 i.insulin_use i.wheelchair_use consultations_lastyear hosp_lastyear phq8_total_score ///

, vce(robust)

vif, uncentered

*Workload-adjusted model: capacity-adjusted model plus clinical variables (caregiver, insulin use, wheelchair use, number of consultations, number	hospitalizations, number of medications, Patient Health Questionnaire-8).

qreg mtbq_tot_stand_score i.departam i.estable_cat i.sex1 i.agecat1 i.est_civil1 i.education1 i.quintiles_assets ///

i.caregiver1 i.insulin_use i.wheelchair_use num_med_total consultations_lastyear hosp_lastyear phq8_total_score ///

, vce(robust)

vif, uncentered

*Note: Database can be accessed through DOI: 10.6084/m9.figshare.28385753*

# References for S1 Appendix

1.	Duncan P, Murphy M, Man MS, Chaplin K, Gaunt D, Salisbury C. Development and validation of the Multimorbidity Treatment Burden Questionnaire (MTBQ). *BMJ Open*. 2018;8(4):e019413. doi:10.1136/bmjopen-2017-019413

2.	Duncan P, Scott LJ, Dawson S, et al. Further development and validation of the Multimorbidity Treatment Burden Questionnaire (MTBQ). *BMJ Open*. 2024;14(4):e080096. doi:10.1136/bmjopen-2023-080096

3.	Dou L, Huang J, Duncan P, Guo L. Translation, cultural adaptation and validation of the Chinese Multimorbidity Treatment Burden Questionnaire(C-MTBQ): a study of older hospital patients. *Health Qual Life Outcomes*. 2020;18(1):194. doi:10.1186/s12955-020-01395-z

4.	Guénette L, Turcotte V, Bélanger L, et al. Multimorbidity Treatment Burden Questionnaire (MTBQ): Translation, cultural adaptation, and validation in French-Canadian. *Can J Aging*. 2023;42(1):126-134. doi:10.1017/S0714980822000058

5.	Schulze J, Breckner A, Duncan P, Scherer M, Pohontsch NJ, Lühmann D. Adaptation and validation of a German version of the Multimorbidity Treatment Burden Questionnaire. *Health Qual Life Outcomes*. 2022;20(1):90. doi:10.1186/s12955-022-01993-z

6.	Hounkpatin HO, Roderick P, Harris S, et al. Change in treatment burden among people with multimorbidity: a follow-up survey. *Br J Gen Pract*. 2022;72(724):e816-e824. doi:10.3399/BJGP.2022.0103

7.	Pedersen MH, Duncan P, Lasgaard M, Friis K, Salisbury C, Breinholt Larsen F. Danish validation of the Multimorbidity Treatment Burden Questionnaire (MTBQ) and findings from a population health survey: a mixed-methods study. *BMJ Open*. 2022;12(1):e055276. doi:10.1136/bmjopen-2021-055276

8.	Engels LWS, van Merode T, Heijmans M, Menting J, Duncan P, Rademakers J. Measurement of treatment burden in patients with multimorbidity in the Netherlands: translation and validation of the Multimorbidity Treatment Burden Questionnaire (NL-MTBQ). *Fam Pract*. 2024;41(6):901-908. doi:10.1093/fampra/cmad100

9.	Vasiliauskienė O, Vasiliauskas D, Duncan P, et al. Validation of the Lithuanian multimorbidity treatment burden questionnaire (MTBQ) and its association with primary care patient characteristics. *Eur J Gen Pract*. 2023;29(1):2284257. doi:10.1080/13814788.2023.2284257

10.	Kroenke K, Strine TW, Spitzer RL, Williams JBW, Berry JT, Mokdad AH. The PHQ-8 as a measure of current depression in the general population. *J Affect Disord*. 2009;114(1-3):163-173. doi:10.1016/j.jad.2008.06.026

11.	Razykov I, Ziegelstein RC, Whooley MA, Thombs BD. The PHQ-9 versus the PHQ-8--is item 9 useful for assessing suicide risk in coronary artery disease patients? Data from the Heart and Soul Study. *J Psychosom Res*. 2012;73(3):163-168. doi:10.1016/j.jpsychores.2012.06.001

12.	Villarreal-Zegarra D, Copez-Lonzoy A, Bernabé-Ortiz A, Melendez-Torres GJ, Bazo-Alvarez JC. Valid group comparisons can be made with the Patient Health Questionnaire (PHQ-9): A measurement invariance study across groups by demographic characteristics. *PLoS One*. 2019;14(9):e0221717. doi:10.1371/journal.pone.0221717

13.	Glasgow RE, Wagner EH, Schaefer J, Mahoney LD, Reid RJ, Greene SM. Development and validation of the Patient Assessment of Chronic Illness Care (PACIC). *Med Care*. 2005;43(5):436-444. doi:10.1097/01.mlr.0000160375.47920.8c

14.	Aragones A, Schaefer EW, Stevens D, Gourevitch MN, Glasgow RE, Shah NR. Validation of the Spanish translation of the Patient Assessment of Chronic Illness Care (PACIC) survey. *Prev Chronic Dis*. 2008;5(4):A113. https://www.ncbi.nlm.nih.gov/pubmed/18793501

15.	ClinicalKey. doi:10.1016/j.aprim.2016.11.008

16.	Garcia-Caselles P, Miralles R, Arellano M, et al. Validation of a modified version of the Gijon’s social-familial evaluation scale (SFES): the “Barcelona SFES Version”, for patients with cognitive impairment. *Arch Gerontol Geriatr Suppl*. 2004;38(9):201-206. doi:10.1016/j.archger.2004.04.028

17.	Miralles R, Sabartés O, Ferrer M, et al. Development and validation of an instrument to predict probability of home discharge from a Geriatric Convalescence Unit in Spain. *J Am Geriatr Soc*. 2003;51:252-257.

18.	EuroQol-a new facility for the measurement of health-related quality of life. *Health Policy*. 1990;16(3):199-208.

19.	Brooks R. EuroQol: the current state of play. *Health Policy*. 1996;37(1):53-72. doi:10.1016/0168-8510(96)00822-6

20.	Rabin R, Charro F de. EQ-SD: a measure of health status from the EuroQol Group. *Ann Med*. 2001;33(5):337-343. doi:10.3109/07853890109002087

21.	Shippee ND, Shah ND, May CR, Mair FS, Montori VM. Cumulative complexity: a functional, patient-centered model of patient complexity can improve research and practice. *J Clin Epidemiol*. 2012;65(10):1041-1051. doi:10.1016/j.jclinepi.2012.05.005

22.	Boehmer KR, Gionfriddo MR, Rodriguez-Gutierrez R, et al. Patient capacity and constraints in the experience of chronic disease: a qualitative systematic review and thematic synthesis. *BMC Fam Pract*. 2016;17(1):127. doi:10.1186/s12875-016-0525-9

23.	May CR, Mair F, Finch T, et al. Development of a theory of implementation and integration: Normalization Process Theory. *Implement Sci*. 2009;4(1):29. doi:10.1186/1748-5908-4-29

24.	May CR, Eton DT, Boehmer K, et al. Rethinking the patient: using Burden of Treatment Theory to understand the changing dynamics of illness. *BMC Health Serv Res*. 2014;14:281. doi:10.1186/1472-6963-14-281
